# Adipocyte hypoxia promotes epithelial-mesenchymal transition-related gene expression and estrogen receptor-negative phenotype in breast cancer cells

**DOI:** 10.3892/or.2015.3880

**Published:** 2015-03-30

**Authors:** AIWEI YAO-BORENGASSER, BEHJATOLAH MONZAVI-KARBASSI, REBECCA A HEDGES, LORA J ROGERS, SUSAN A KADLUBAR, THOMAS KIEBER-EMMONS

**Affiliations:** 1 Division of Medical Genetics, University of Arkansas for Medical Sciences, Little Rock, AR 72205, USA; 2 Department of Pathology, College of Medicine, and Winthrop P. Rockefeller Cancer Institute, University of Arkansas for Medical Sciences, Little Rock, AR 72205, USA

**Keywords:** breast cancer, adipocytes, HIF1α, estrogen receptor, epithelial-mesenchymal transition

## Abstract

The development of breast cancer is linked to the loss of estrogen receptor (ER) during the course of tumor progression, resulting in loss of responsiveness to hormonal treatment. The mechanisms underlying dynamic ERα gene expression change in breast cancer remain unclear. A range of physiological and biological changes, including increased adipose tissue hypoxia, accompanies obesity. Hypoxia in adipocytes can establish a pro-malignancy environment in breast tissues. Epidemiological studies have linked obesity with basal-like breast cancer risk and poor disease outcome, suggesting that obesity may affect the tumor phenotype by skewing the microenvironment toward support of more aggressive tumor phenotypes. In the present study, human SGBS adipocytes were co-cultured with ER-positive MCF7 cells for 24 h. After co-culture, HIF1α, TGF-β, and lectin-type oxidized LDL receptor 1 (LOX1) mRNA levels in the SGBS cells were increased. Expression levels of the epithelial-mesenchymal transition (EMT)-inducing transcription factors FOXC2 and TWIST1 were increased in the co-cultured MCF7 cells. In addition, the E-cadherin mRNA level was decreased, while the N-cadherin mRNA level was increased in the co-cultured MCF7 cells. ERα mRNA levels were significantly repressed in the co-cultured MCF7 cells. ERα gene expression in the MCF7 cells was decreased due to increased HIF1α in the SGBS cells. These results suggest that adipocytes can modify breast cancer cell ER gene expression through hypoxia and also can promote EMT processes in breast cancer cells, supporting an important role of obesity in aggressive breast cancer development.

## Introduction

Metastasis is the leading cause of breast cancer-related deaths even though early-stage breast cancers are not life threatening. The majority of breast cancer patients exhibit an estrogen receptor α (ERα)-positive phenotype (1,2), and ERα levels are increased in malignant lesions (3). ERα-positive tumors are responsive to adjuvant hormonal therapy, and are associated with improved patient survival compared to patients with more aggressive ERα-negative tumors (4). However, the development of breast cancer is linked to the loss of ERα during the course of tumor progression, resulting in loss of responsiveness to hormonal treatment (5). The mechanisms underlying dynamic ERα gene expression change in breast cancer are not clear. Several *in vitro* studies have shown that hypoxic conditions could lead to downregulation of ERα gene expression and to the increase in ERα protein degradation in human breast cancer cells (6–9).

A wide range of physiological and biological changes, including increased adipose tissue hypoxia and oxidative stress, accompanies obesity. The hypoxic state of obese adipose tissue could be related to the failure of vascular growth required for tissue expansion and decreased oxygen diffusion over longer distances due to increased cell size (10–13). Also, metabolism of excess free fatty acids in obesity by the mitochondrion results in increased generation of reactive oxygen species (ROS) (14,15). Obesity-induced ROS production, mainly generated by NADPH oxidase, leads to the elevation of systemic oxidative stress, as well as dysregulated production of adipokines in adipocytes (10). Both hypoxia and oxidative stress affect the production of many adipocyte-derived proteins involved in angiogenesis, inflammation and extracellular matrix remodeling. These events establish a pro-malignancy environment in breast tissue. Several population studies have shown that obesity is a risk factor in basal-like cancer development. Studies found that increased waist-to-hip ratio and waist circumference, two surrogates for abdominal adiposity, were associated with a strong increase in the risk of basal-like cancer among both pre- and post-menopausal women (16,17). A recent study showed that metabolic syndrome, characterized by obesity and insulin resistance, is associated with ER/PR and HER-2 triple-negative breast cancer (18). Using a two-dimensional co-culture system, Dirat *et al* (19) demonstrated that murine and human breast cancer cells co-cultured with murine adipocytes showed increased invasive capacities *in vitro* and *in vivo*. These data suggest that cancer-associated adipoctyes (CAAs) are essential for breast tumor development and progression. The mechanisms responsible for the effect of adipocytes on tumorigenesis at the molecular level are largely unknown. We hypothesized that the function of CAAs is dependent, at least partly, on their interaction with invasive cancer cells. The aim of the present study was to investigate the putative changes in gene expression profiles in adipocytes and breast cancer cells that have been co-cultured. The data suggest that adipocytes co-cultured with cancer cells downregulate ER gene expression and promote epithelial-mesenchymal transition (EMT) in breast cancer cells through upregulation of HIF1α.

## Materials and methods

### Cell culture

The human breast cancer cell line MCF7 was purchased from the American Type Culture Collection (ATCC) and maintained following the protocol described by ATCC at 37°C in an incubator containing 5% CO_2_. MCF7 cells were cultured in improved Dulbecco’s modified Eagle's medium (DMEM) with 10% FBS and 0.01 mg/ml insulin (both from Life Technologies, Carlsbad, CA, USA).

Human adipocyte cells, derived from the stromal vascular fraction of an infant with Simpson-Golabi-Behmel syndrome (SGBS) were provided by Dr Wabitsch (20). SGBS cells were maintained and differentiated into adipocytes, as described previously (20). Oil red O staining, and the detection of adipocyte-specific mRNA and/or protein expression assessed differentiation.

### MCF7 and SGBS cell co-culture

**Figure 1:**
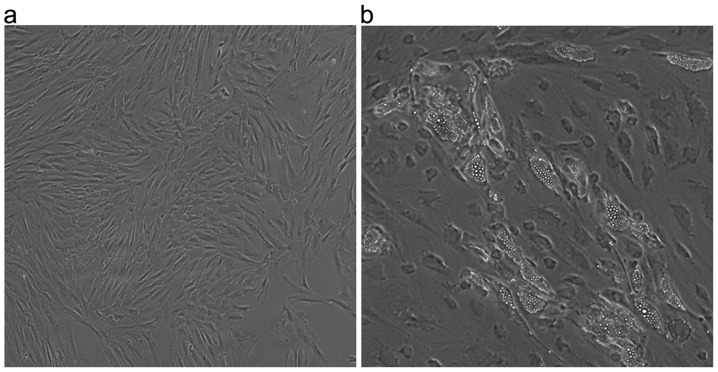
Images show pre-differentiated SGBS cells (a) and post-differentiated SGBS adipocytes (b).

**Figure 2:**
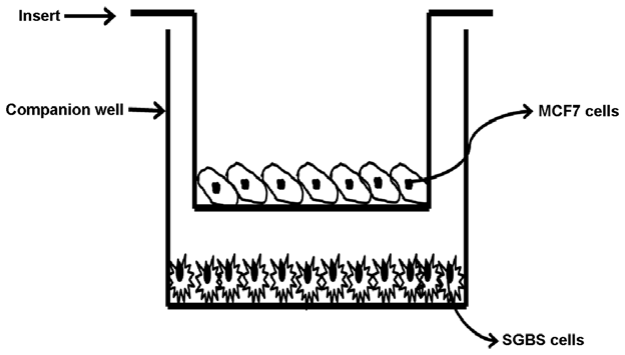
Scheme of the co-culture experiments. Pre-adipocytes, SGBS, were seeded in the wells overnight to allow cells to adhere. Adherent cells were differentiated for 8 days in a differentiation cocktail medium. MCF7 cells were seeded on polyester membrane inserts overnight before the co-culture, which was started by transferring the inserts into the SGBS-containing wells.

Breast cancer cell and adipocyte co-culture experiments were performed using a modification of a previously described method (21). Briefly, 200,000 undifferentiated SGBS cells were seeded in the wells of a 6-well companion overnight, followed by differentiation induction for 8 days. Fig. 1 is a representative image of the pre- and post-differentiated SGBS cells. One-half-million MCF7 cells were seeded on polyester membrane inserts in 6-well culture dishes with 0.4-*μ*m pore size and pore density 4×10^6^/cm^2^ overnight before co-culture with the adipocytes. The co-culture was assembled when the adipocytes were at least 60% differentiated. The adipocytes and tumor cells were separated by 0.9 mm (membrane to bottom of well) in the same well with free exchange of medium. Fig. 2 is a scheme of the co-culture system. The SGBS and MCF7 cells were co-cultured for 24 h along with individual controls of SGBS and MCF7 cells cultured alone. Co-culture experiments were performed in triplicate. Following co-culture, the cells from the inserts (MCF7 cells) and wells [SGBS or HIF1α siRNA (siHIF1α)-transfected SGBS cells] were collected separately, and then lysed with RNA lysis buffer from Life Technologies.

### HIF1α siRNA treatment of adipocytes

Differentiated SGBS cells were transfected with 30 nM of siHIF1α or negative control siRNA using siPORT™ Amine transfection reagent (both from Life Technologies) following the manufacturer's instructions. Total RNA was isolated after a 48-h transfection as described below. The percentage of knockdown target gene expression was determined using quantitative real-time RT-PCR.

### RNA isolation and real-time RT-PCR

Total RNA from the cell culture was isolated using RNAqueous kit (Applied Biosystems). The quantity and quality of the isolated RNA were determined using Agilent 2100 Bioanalyzer (Agilent Technologies, Inc., Palo Alto, CA, USA). Real-time RT-PCR was performed as described previously (22). Briefly, 1 *μ*g of total RNA was reverse-transcribed using random hexamer primers with TaqMan reverse transcription reagents (Applied Biosystems). Reverse-transcribed RNA was amplified with SYBR-Green PCR Master Mix (Applied Biosystems) plus 0.3 *μ*M of gene-specific upstream and downstream primers during 40 cycles on an Applied Biosystems 7900HT Fast Real-Time cycler. The 2^−ΔΔCT^ method was used to assess the target transcript in a treatment group relative to that of an untreated control group using expression of an internal control 18S to normalize data (23). Each cycle consisted of denaturation at 95°C for 15 sec, and annealing and extension at 60°C for 60 sec. The primer sequences are shown in Table I.

### Statistical analysis

Paired t-tests were used to compare baseline and treatment measurements within a group. All data from samples are expressed as mean ± SEM.

## Results

### Gene expression level changes in human adipocytes co-cultured with breast cancer cells

**Figure 3:**
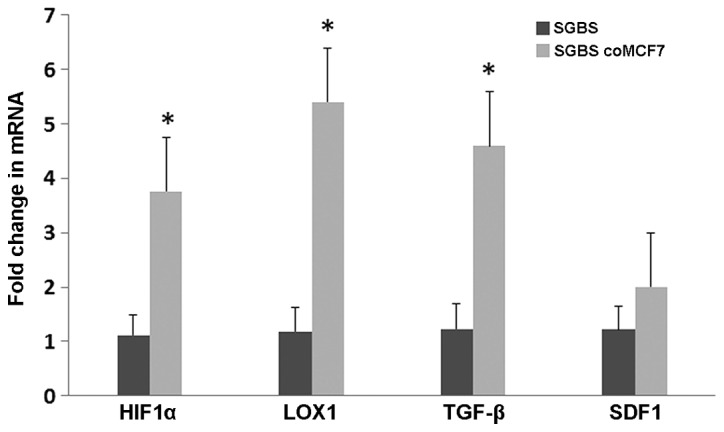
Expression of selected genes in the SGBS adipocytes co-cultured with the MCF7 cells. Differentiated SGBS cells were co-cultured with MCF7 cells for 24 h as described in Materials and methods. The mRNA levels of HIF1α, LOX1, TGF-β, and SDF1 from the co-cultured SGBS and control SGBS cells were determined by RT-PCR. The data were normalized with 18S RNA level and analyzed with the 2^−ΔΔCT^ method, ^*^P<0.05.

In order to study the interaction between adipocytes and breast tumor cells and its impact on hormone receptor status and aggressive phenotype, human breast cancer ER-positive MCF7 cells were co-cultured with differentiated human SGBS adipocytes for 24 h using the method described in Materials and methods. The transfection did not affect SGBS cell growth and differentiation. There were no changes observed in cell morphology or purity in either the MCF7 or SGBS cells after co-culture. Gene expression levels of HIF1α were determined with RT-PCR in both the SGBS and MCF7 cells. As shown in Fig. 3, the HIF1α mRNA level in the SGBS adipocytes was increased >3-fold after co-culture with the MCF7 cells (P<0.05). The co-culture had no effect on the expression levels of HIF1α in the MCF7 cells (data not shown).

We also examined the expression levels of genes related to cancer-promoting factors. Fig. 3 shows that the expression levels of inflammatory gene lectin-type oxidized LDL receptor 1 (LOX1) and TGF-β were increased in the SGBS cells co-cultured with the MCF7 cells, while stromal cell-derived factor 1 (SDF1) remained the same.

We also co-cultured the SGBS cells with ER-negative MDA-MB-231 and transformed epithelial MCF-7-10A cells. There were no similar changes detected in those co-cultured cells (data not shown).

### Human adipocytes decrease MCF7 ERα gene expression and this event is adipocyte HIF1α-dependent

**Figure 4:**
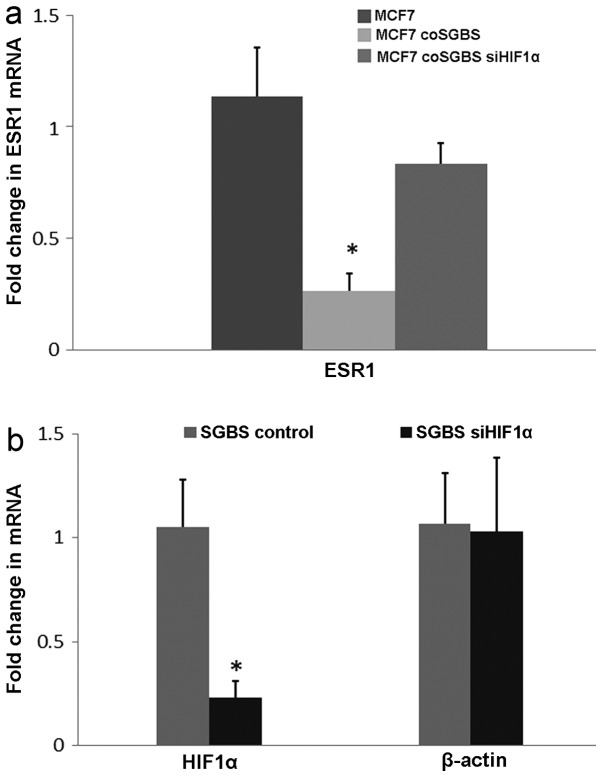
(a) ESR1 mRNA levels in MCF7, MCF7 co-cultured with SGBS (MCF7 coSGBS) and MCF7 cells co-cultured with SGBS that had been transfected with siRNA against HIF1α (MCF7 coSGBS siHIF1α). Total RNA was extracted for gene expression analysis (n=3 for each mRNA expression level). ^*^P<0.05. (b) Differentiated SGBS cells were treated with siHIF1α as described in Materials and methods. HIF1α knockdown efficiency was determined with RT-PCR in the SGBS cells, ^*^P<0.05. The data were normalized with the 18S RNA level and analyzed with the 2^−ΔΔCT^ method.

Other investigators have shown that ERα gene expression and protein levels are downregulated by HIF1α when breast cancer cells are cultured under hypoxic conditions (6–9). Since increased HIF1α was observed in the SGBS cells co-cultured with the MCF7 cells, the ERα mRNA level, encoded by ESR1, in the MCF7 cells co-cultured with the SGBS cells was determined. As shown in Fig. 4a, ESR1 gene expression was significantly repressed >75% (P=0.02) in the MCF7 cells co-cultured with the SGBS cells (MCF7 coSGBS), compared to that of the control MCF7 cells.

To study the mechanism of adipocytes on ERα expression, SGBS cells were transfected with 30 nM of HIF1α siRNA (siHIF1α) for 48 h before co-culture with the MCF7 cells. The percentage of knockdown of HIF1α expression was determined using real-time RT-PCR. The expression of HIF1α in the siHIF1α-transfected cells decreased ~70%, while β-actin expression was comparable to the control levels (Fig. 4b). The ESR1 gene expression level in the MCF7 cells was not affected by the adipocytes that had been transfected with siHIF1α (MCF7 coSGBS siHIF1α, Fig. 4a).

### Human adipocytes regulate the gene expression of EMT-inducing factors in MCF7 cells

**Figure 5:**
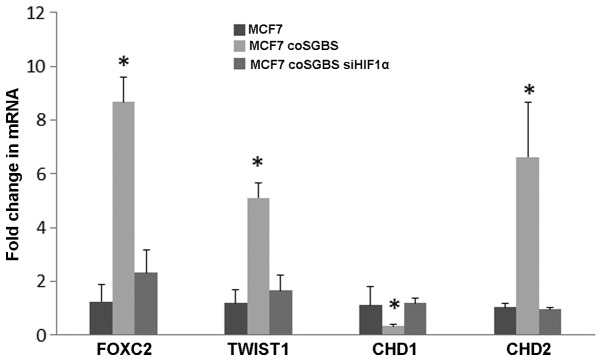
Effects of SGBS adipocytes on EMT-inducing gene expression in MCF7 cells. SGBS or SGBS transfected with siHIF1α were co-cultured with MCF7 cells. Gene expression levels of FOXC2, TWIST1, CHD1 and CHD2 were determined in the co-cultured MCF7 cells using real-time RT-PCR. The data were normalized with the 18S RNA level and analyzed with the 2^−ΔΔCT^ method. ^*^P<0.05 compared to the control MCF7 cells.

When co-cultured with murine adipocytes 3T3, human low-invasive breast cancer cells ZR75 exhibited an enhanced invasive phenotype (19). We compared the EMT-related gene expression levels before and after MCF7 cells were co-cultured with the SGBS adipocytes. As shown in Fig. 5, expression levels of EMT-inducing transcription factors FOXC2 and TWIST1 were significantly increased (5- and 8-fold, respectively) after co-culture with SGBS. E-cadherin mRNA level, encoded by CDH1, was decreased 70%, while the N-cadherin mRNA level, encoded by CDH2, increased >6-fold in the co-cultured MCF7 cells. However, there was no gene expression level change detected between the MCF7 and MCF7 cells co-cultured with the siHIF1α-treated SGBS cells (Fig. 5).

## Discussion

In the present *in vitro* study, we demonstrated the interaction between human breast cancer cells and human adipocytes when they were co-cultured. MCF7 cells increased HIF1α gene expression in the SGBS cells. Conversely, downregulation of HIF1α in the SGBS cells was accompanied by a decrease in *ESR1* gene expression in the MCF7 cells. The mRNA levels of genes that promote the EMT process were also increased in the MCF7 cells after co-culture with the adipocytes.

Several studies have shown that hypoxic conditions repress ERα mRNA and protein levels in breast cancer cells (6,7,9,24). Kurebayashi *et al* (7) demonstrated that the ERα protein level was significantly lower in nuclear HIF1α-positive breast tumors than the level in negative tumors. Ryu *et al* (6)using ER-positive T47-D cells demonstrated that ERα mRNA and protein levels were degraded under hypoxic conditions. In breast cancer patients, obesity is consistently linked to reduced survival and high recurrence rate regardless of menopausal status (25–27).

While the mechanisms underlying the link are largely unknown, studies have shown that obesity in breast cancer patients have an impact on the gene expression patterns of tumors. Creighton *et al* (28) demonstrated that obese breast cancer patient tumors possess a gene transcription signature of increased IGF signaling pathway with low levels of ER. Our co-culture and siHIF1α transfection data suggest that HIF1α generated from adipocytes co-cultured with MCF-7 cells downregulated ER gene expression in the breast cancer cell line. Consistent with these data, Ryu *et al* (6) demonstrated that ESR1 expression is dependent on HIFα. These findings support our statement that changes in the HIF1α mRNA level can lead to the gene expression regulation of ERS1. This provides direct evidence that adipocytes interact with tumor cells, creating a hypoxic environment resulting in reduced ER expression.

Consistent with our findings, a recent study showed that both human and mouse breast cancer cells co-cultured with murine adipocytes exhibited an enhanced invasive phenotype (19). Meanwhile, adipocytes co-cultured with breast cancer cells exhibited increased expression of proteases such as matrix metalloproteinase-11 and pro-inflammatory cytokines, such as IL-6. In addition, E-cadherin mRNA and protein levels in those co-cultured cells were decreased. Our data further support these findings by demonstrating that EMT-related gene expression was increased and E-cadherin mRNA was decreased in human breast cancer cells co-cultured with human adipocytes. The loss of E-cadherin, a critical component for cell adhesion, is a key event in dissolution of cell-cell contacts during EMT. In line with decreased E-cadherin expression, gene expression of EMT-promoting factors such as FOXC2, TWIST1 and N-cadherin were increased in the co-cultured MCF7 cells.

Our data also demonstrated an increase in TGF-β and LOX1 gene expression in the SGBS cells co-cultured with the MCF7 cells. TGF-β is an established inducer of EMT in breast cancer progression (reviewed in ref. 29). LOX1, a scavenger receptor for oxidized LDL, has been shown to stimulate the migration of MDA-MB-231 breast cancer cells (30) and to promote the EMT process in kidney epithelial cell line NRK52E (31). In our system, the TGFβ gene expression level did not change after siHIF1α transfection in the SGBS cells (data not shown), suggesting that TGFβ expression by SGBS cells is not regulated by HIF1α. However, HIF1α may exert its function by cross-talking with TGFβ or its downstream signaling molecules such as Smad3/4. Studies have shown that HIF1α and TGFβ pathways (Smad3/4) interact with each other to regulate expression of the HIF1α-responsive gene, erythropoietin (32).

Other HIF1α-responsive genes such as VEGF and basic fibroblast growth factor (bFGF) could be potential factors that regulate EMT gene expression in MCF7 cells (33,34). The present study did not explore the soluble factors derived from MCF7 cells that stimulated hypoxia response in the SGBS cells. However TNFα, TGFβ and IL-6 are potential candidates to be examined in future studies. The increase in TGFβ and LOX1 gene expression also suggests that the inflammatory and TGFβ pathway could also cross-talk with HIF1α regulation. Studies have demonstrated that the HIF1α promoter contains a functional NF-κB binding site (35). We did not investigate the mechanisms controlling HIF1α, and it is possible that NF-κB plays a role in the regulation of HIF1α expression. This possibility will be explored in future experiments.

Using a human adipocyte and breast cancer cell line co-culture system, the present study supports the hypothesis that close interactions exist between tumor cells and adipocytes. Tumor cells stimulate hypoxia in adipocytes, which, in turn, enhance more invasive gene expression in tumor cells. The present study provides a foundation for future clinical and cellular studies in order to determine the impact of obesity in breast cancer development.

In summary, adipocytes can modify ER gene expression through hypoxia and also can promote EMT processes in breast cancer cells, supporting an important role of obesity in the development of an aggressive breast cancer phenotype.

## Figures and Tables

**Table I:** Primer sequences used for PCR.

Gene	Forward	Reverse
18S	TTCGAACGTCTGCCCTATCAA	ATGGTAGGCACGGCGACTA
β-actin	CGCTGCCAGCTCTCGCACTC	TTGCGACCGGCAGAGAAACGC
HIF1α	TGCTCATCAGTTGCCACTTC	CAAATCACCAGCATCCAGAA
ESR1	AGGTGGGATACGAAAAGACCG	AAGGTTGGCAGCTCTCATGTC
TWIST1	GGAGGATGGAGGGGGCCTGG	ATGACATCTAGGTCTCCGGCCCTG
FOXO2	CCACGCAGCCCCCTACTCCT	GCTGGGAAGCGAAGCCGGAG
CDH1	ACGCCTGGGACTCCACCTACA	AACGGAGGCCTGATGGGGCG
CDH2	GCTGTCGGTGACAAAGCCCCT	TGCCCTCAAATGAAACCGGGCT
LOX1	CTCCTTTGATGCCCCACTTA	TTTCCGCATAAACAGCTCCT
TGF-β	GTGGAAACCCACAACGAAAT	CGGAGCTCTGATGTGTTGAA
SDF1	CCAAACTGTGCCCTTCAGAT	CTTTAGCTTCGGGTCAATGC
